# Pentavalent Antimonials: New Perspectives for Old Drugs

**DOI:** 10.3390/molecules14072317

**Published:** 2009-06-30

**Authors:** Frédéric Frézard, Cynthia Demicheli, Raul R. Ribeiro

**Affiliations:** 1 Departamento de Fisiologia e Biofísica, Instituto de Ciências Biológicas, Universidade Federal de Minas Gerais, Av. Antônio Carlos 6627, Pampulha, 31270-901 Belo Horizonte, MG, Brazil; E-mail: raulrio@ufmg.br (R.R.); 2 Departamento de Química, Instituto de Ciências Exatas, Universidade Federal de Minas Gerais, Av. Antônio Carlos 6627, Pampulha, 31270-901 Belo Horizonte, MG, Brazil; E-mail: demichel@netuno.lcc.ufmg.br (C.D.)

**Keywords:** pentavalent antimonials, meglumine antimoniate, cyclodextrin, liposomes, leishmaniasis

## Abstract

Pentavalent antimonials, including meglumine antimoniate and sodium stibogluconate, have been used for more than half a century in the therapy of the parasitic disease leishmaniasis. Even though antimonials are still the first-line drugs, they exhibit several limitations, including severe side effects, the need for daily parenteral administration and drug resistance. The molecular structure of antimonials, their metabolism and mechanism of action are still being investigated. Some recent studies suggest that pentavalent antimony acts as a prodrug that is converted to active and more toxic trivalent antimony. Other works support the direct involvement of pentavalent antimony. Recent data suggest that the biomolecules, thiols and ribonucleosides, may mediate the actions of these drugs. This review will summarize the progress to date on the chemistry and biochemistry of pentavalent antimony. It will also present the most recent works being done to improve antimonial chemotherapy. These works include the development of simple synthetic methods for pentavalent antimonials, liposome-based formulations for targeting the *Leishmania* parasites responsible for visceral leishmaniasis and cyclodextrin-based formulations to promote the oral delivery of antimony.

## Introduction

Antimony has been used in therapeutics for several centuries. Some authors have suggested its earliest use in ancient Egypt for cosmetic purposes. However, it has been shown that this statement was based on a misreading of the ancient texts [1,2,3]. The importance of antimony in the early medicine is well documented, due to the debate created around their utilization in this period [4].

The most significant clinical use of organoantimonials during the last century is certainly that in the treatment of leishmaniases. Leishmaniases are infective parasitic diseases, which are endemic in 88 countries, 22 in the New World and 66 in the Old World, and affect mainly poor and marginalized populations [5]. These clinical manifestations of the disease can involve the skin, with local (cutaneous), diffuse (diffuse cutaneous) or disfiguring lesions (mucocutaneous), or the viscera, leading to death if untreated. It is caused by parasitic protozoa of the genus *Leishmania*, transmitted to humans via the bite of sandflies. Wild and domesticated animals, and humans themselves can act as a reservoir of infection. *Leishmania* parasite is found as a motile promastigote in the sandfly, it transforms into an amastigote when engulfed by host macrophages, and resides in the acidic environment of secondary lysosomes [5].

At the beginning of the last century, Gaspar Vianna, pioneer researcher in the treatment of leishmaniasis, reported the efficacy of antimony(III) potassium tartrate (tartar emetic) for treatment of muco-cutaneous leishmaniasis [6]. This activity was confirmed in visceral leishmaniasis in Italy [7], Africa [8] and India [9]. Later, the clinical use of this compound was interrupted because of severe side-effects.

The less toxic pentavalent antimony (Sb(V)) complexes were introduced in the therapeutics of leishmaniases from the 1940s. Even though pentavalent antimonials are still the first-line drugs against all forms of leishmaniasis, their use in the clinical setting has several limitations [10,11]. These compounds have to be given parenterally, daily, for at least three weeks (typically, 20 mg of Sb/kg/day for 20–30 days). Antimony therapy is often accompanied by local pain during intramuscular injections and by systemic side effects, requiring very careful medical supervision. Typical side-effects include nausea, vomiting, weakness and myalgia, abdominal colic, diarrhea, skin rashes and hepatotoxicity, together with the most important cardiotoxicity. The appearance of drug resistance is another important problem in the treatment of this disease [12]. Second-line drugs (pentamidine and amphotericin B) are also limited by severe side effects and the need for parenteral administration [11]. All these factors contribute to compliance difficulties and treatment failures. In the light of these limitations, the World Health Organization strongly recommends and supports research into new drugs against leishmaniasis [13]. However, the lack of any significant commercial return for the neglected diseases, such as leishmaniasis, has resulted in insufficient funding and commitment from both public sector agencies and the pharmaceutical industry, for drug research and development [13]. In this context, strategies based on the improvement of existing drugs have been more successful than those based on the design of new chemical entities. Advances include the development of more effective and safer formulations for existing anti-leishmanial drugs, the use of drugs originally designed and evaluated for nonrelated diseases, novel drug combinations and therapeutic protocols [14]. Much effort was also devoted to the development of oral and topical drug formulations. As main achievements, two new drugs have recently reached the market: a liposomal formulation of amphotericin B (AmBisome^®^, NeXstar Pharmaceuticals) [15] and miltefosine (Impavido^®^, Zentaris), originally developed as anticancer drugs, for oral treatment of visceral leishmaniasis [16]. Both drugs produced remarkable cure rates (higher than 90%) in clinical trials against visceral leishmaniasis, but also presented some limitations. The high cost of AmBisome^®^ makes its large-scale use in developing countries problematic. On the other hand, miltefosine was found teratogen to animals and exhibits a rather narrow therapeutic window in clinical trials [16].

In the specific case of pentavalent antimonial drugs, recent advances include the development of liposome- and cyclodextrin-based formulations for improved drug bioavailability, new insights into their chemistry and mechanism of action that may result in novel strategies for improved treatment.

Indeed, until recently, little was known about the chemical structure of these compounds and the methods used in the industry for their preparation [11,17]. As a consequence, inadequate manufacture has already occurred, as evidenced by the serious side effects produced by some commercial forms of pentavalent organoantimonials [18,19]. 

It is although noteworthy that recent studies with pentavalent antimonials have revealed their effectiveness in experimental models of cancer, hepatitis C and AIDS [20]. This context explains the renewed interest in the chemistry and biochemistry of these old drugs, as well as in the development of more effective pharmaceutical formulations. 

This review will cover the progress recently achieved in the chemistry and biochemistry of pentavalent antimonials, as well as some promising liposome- and cyclodextrin-based pharmaceutical formulations. The perspectives for drug design will also be discussed.

## Structure and Mechanism of Action

The two main antimonials, under current clinical use, are complexes of Sb(V) with *N*-methyl-D-glucamine (meglumine antimoniate or Glucantime^®^) or sodium gluconate (sodium stibogluconate or Pentostam^®^). Although the exact structure of these complexes remained unknown for decades, mainly because of the amorphous state of these compounds, the use of mass spectrometric approaches and NMR techniques has allowed significant progress in this area.

Fast-atom bombardment mass spectrometric (FAB-MS) data on the commercially available meglumine antimoniate suggested a main structure in which two molecules of meglumine (NMG) coordinate with a single Sb atom in a symmetrical geometry [21]. On the other hand, positive ion electrospray ionization mass spectrometry (ESI(+)-MS) analyses have suggested the existence of a mixture of polymeric structures with the general formula (NMG-Sb)_n_-NMG [22]. Further analyses of meglumine antimoniate by ESI-MS, in both the positive and negative modes, showed negatively-charged 1:1 (*m/z* 364) and 2:2 (*m/z* 765) Sb(V)–meglumine complexes and supported the predominance of zwitterionic species in solution [23]. ES-MS measurements of sodium stibogluconate also showed that it consists of a mixture of oligomeric structures [23], in agreement with earlier results obtained by molecular sieve chromatography [24], and consistent with the general formula for meglumine antimoniate ((GLU-Sb)*n*-GLU and (GLU-Sb)*n* (GLU, gluconate). Osmolarity measurements suggested the predominance of the 1:1 Sb-NMG and Sb-SSG complexes in diluted samples [23]. This interpretation was further supported by HPLC-inductively coupled plasma-MS and ESI-MS analyses in the case of sodium stibogluconate [25].

**Figure 1:**
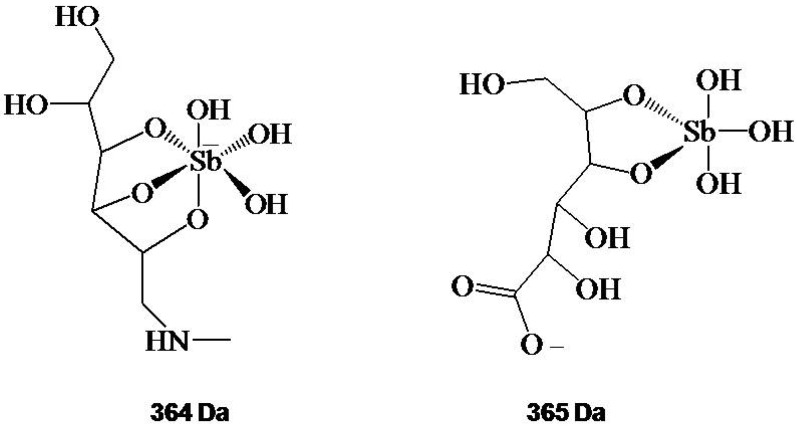
Proposed structural formula for 364 Da and 365 Da ions identified by ESI(-)-MS in aqueous solutions of meglumine antimoniate and stibogluconate, respectively. Adapted from Ref. [23].

A proton NMR study allowed the assignment of proton resonances in meglumine antimoniate, and established the existence of two distinct environments for NMG [26]. Comparison of these resonances to those of the free NMG ligand indicated that NMG molecules coordinate Sb in two different fashions, and suggested either the coexistence of at least two different complexes or the existence of a major complex in which two NMG molecules are coordinated with Sb in an asymmetrical geometry [26]. It is noteworthy that MS and NMR data obtained so far are expected to be useful to the quality control of meglumine antimoniate, following industrial production. Figure 1 shows the structures actually proposed for the predominant Sb-ligand complex in diluted solutions of meglumine antimoniate and sodium stibogluconate.

**Figure 2:**
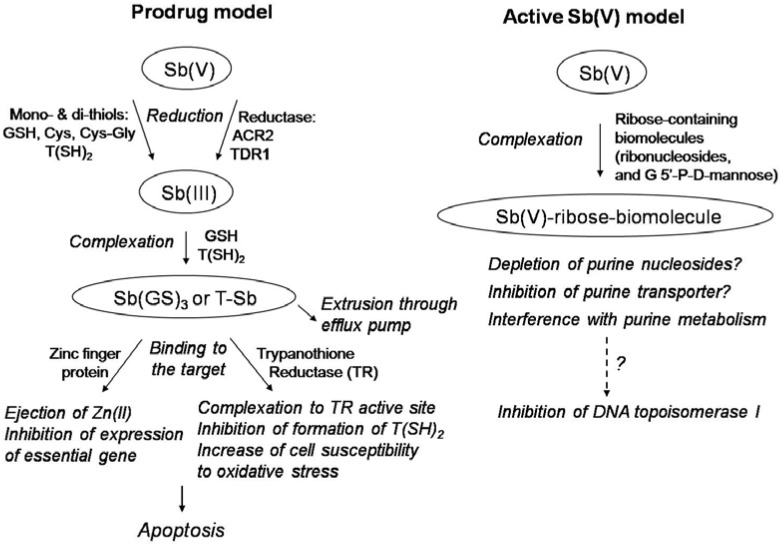
Two main models proposed for the mechanism of action of pentavalent antimonials against leishmaniasis. According to the “Prodrug model”, Sb(V) is reduced to Sb(III) in order to exert antileishmanial activity. According to the “Active Sb(V) model”, Sb(V) exhibits intrinsic antileishmanial activity.

The mode of action of pentavalent antimonials against leishmaniasis still remains poorly understood [3,4]. It is not clear whether the final active form of pentavalent antimonials is Sb(V) or Sb(III). Figure 2 illustrates the two main models proposed so far for the mechanism of action of pentavalent antimonials.

According to the first model, Sb(V) would behave as a prodrug, that is reduced within the organism into more toxic and active Sb(III). This model is supported by the observation that part of Sb(V) is reduced *in vivo* into more toxic Sb(III) [27,28,29].

Recent studies also indicated that thiols may act as a reducing agent in this conversion [30,31,32]. Four different thiols have been evaluated: glutathione (GSH), which is the main thiol in the cytosol of mammalian cells; cysteine (Cys) and cysteinyl-glycine (Cys-Gly), which are the predominant thiols within lysosomes [33,34], and the glutathione-spermine conjugate, trypanothione (T(SH)_2_), which is the predominant thiol within the parasite [35]. It has been reported that Cys, Cys-Gly and T(SH)_2_ do promote the reduction of Sb(V) into Sb(III) at 37ºC. Strikingly, the initial rates of reduction of Sb(V) were much greater in the presence of Cys-Gly, Cys and T(SH)_2_, than in the presence of GSH [30]. These reactions occurred at both pH 5 and pH 7, but were favored at acidic pH and slightly elevated temperature. These data support the hypothesis that Sb(V) is reduced *in vivo* by T(SH)_2_ within *Leishmania* parasites and by Cys or Cys-Gly within the acidic compartments of mammalian cells. Thus, reduction was found to occur preferentially in amastigotes [29], which have a lower intracellular pH and live at a higher temperature than promastigotes. On the other hand, recent studies have suggested the participation of an parasite-specific enzyme in the process of reduction of Sb(V) to Sb(III), thiol-dependent reductase (TDR1) [36] and/or antimoniate reductase (ACR2) [37].

Evidence was obtained that Sb(III) enters *Leishmania* cells primarily though an aquaglyceroporin named AQP1 and that the level of expression of this transporter may modulate the resistance of the parasite to Sb(III) [38].

Sb(III) is classified as a borderline metal ion and has a high affinity towards nitrogen- and sulfhydryl-containing ligands. The anti-leishmanial mechanisms of Sb(III) is probably related to its interaction with sulfhydryl-containing biomolecules, including thiols, peptides, proteins and enzymes.

Thus, Sb(III) was found to form stable complex with the major intracellular thiols, GSH and T(SH)_2_, in the form of a 1:3 and 1:1 Sb-thiol complexes, respectively [31,32,39]. These abundant low molecular mass thiols, however, are rather metal carriers than final molecular targets leading to cell death. Once Sb(III) is in the cell and is conjugated to T(SH)_2_, the complex can be sequestered inside a vacuole or extruded by ATP-binding cassette (ABC) transporters [40,41]. Thus, increase in total thiols (Cys, GSH and T(SH)_2_) and/or overexpression of ABC transporter are often observed in metal-resistant *Leishmania*. The thiol increase is mediated by the overexpression or amplification of a number of different genes involved in the synthesis of GSH or polyamines, which are the two building blocks of T(SH)_2_ [42]. This suggests that if one could lower T(SH)_2_ levels in the cell, it might be possible to reduce resistance. Interestingly, resistance to Sb(V) in *Leishmania donovani* clinical isolates was reversed in animal models by treatment with an inhibitor of GSH biosynthesis [43], suggesting a treatment combination that could revert resistance.

Among the potential molecular targets of Sb(III), evidence was obtained that trypanothione reductase (TR) or zinc-finger protein may be involved. Such interaction is consistent with the modality of Cys binding of thiophilic metals such as As(III), Sb(III), and Bi(III). Metal-bound Cys are fully deprotonated thiolate anions, the nucleophilicity of which is greatly attenuated upon formation of metal complexes with high thermodynamic stability.

The trypanothione/TR system, which keeps T(SH)_2_ under the reduced state, replaces the nearly ubiquitous glutathione/GR system, protects trypanosomatids from oxidative damage and toxic heavy metals and delivers the reducing equivalents for DNA synthesis [44]. Although TR shares structural and mechanistic similarity with GR, differences in the disulfide binding site between TR and GR draw the interest for selective inhibition. Fairlamb and co-workers recently reported that trivalent antimonials interfere with T(SH)_2_ metabolism by inhibiting TR and inducing rapid efflux of intracellular T(SH)_2_ and GSH in intact *Leishmania* cells [45,46]. The crystal structures of the complex of TR with NADPH and Sb(III) in the reduced state was recently described [47]. Sb(III), was found to be coordinated by the two redox-active catalytic cysteine residues (Cys52 and Cys57), one threonine residue (Thr335), and His461′ of the 2-fold symmetry related subunit in the dimer.

In another recent study, our group reported the ability of Sb(III) to bind to a CCHC zinc finger peptide model and to promote the ejection of Zn(II) [48]. The zinc finger domain is characterized by the coordination of a zinc atom by several amino acid residues, usually cysteines and histidines. These structural elements are associated with protein–nucleic acid and protein–protein interactions as well as extraordinarily diverse functions, including DNA recognition, RNA packaging, protein folding and assembly, lipid binding, transcriptional activation, cell differentiation and growth and regulation of apoptosis [49]. Several zinc finger proteins sharing the CCHC motif have been identified in trypanosomatids and have been shown to be involved in different cellular functions. In *Leishmania major*, the protein HEXBP, containing nine CCHC motifs, binds to the hexanucleotide repeat sequence found in the intervening region of the GP63 gene cluster, the most abundant surface glycoprotein of this protozoan, and it is likely to be involved in DNA replication, structure and repair [50].

Treatment of *Leishmania infantum* amastigotes with Sb(III) at low concentrations was found to induce DNA fragmentation, suggesting the appearance of late events of programmed cell death (apoptosis) [51]. Further studies on the mechanisms of the apoptotic cell death pathway in *Leishmania* indicated that intracellular Ca^2+^ plays a central role in intracellular parasite clearance [52], a phenomenon previously documented in the oxidative-stress-induced apoptosis-like death in *Leishmania donovani* promastigotes [53]. 

According to the second model, Sb(V) would present intrinsic anti-leishmanial activity. Sodium stibogluconate, but not Sb(III), was shown to specifically inhibit type I DNA topoisomerase from *Leishmania donovani* through the inhibition of the unwinding and cleavage of the supercoiled plasmid pBR322, and to stabilize topoisomerase and DNA covalent complexes but not calf-thymus topoisomerase I and *Escherichia coli* DNA gyrase [54,55]. Furthermore, the *in vivo* sensitivity and resistance of *Leishmania* was correlated with the effect of such a complex [56].

Demicheli and co-workers have reported the formation of a complex between adenine ribonucleoside and Sb(V) [57]. This was the first report of a physiologically-relevant biomolecule capable of forming stable complexes with Sb(V). Both 1:1 and 1:2 Sb(V)-ribonucleoside complexes were evidenced [57,58,59]. The large changes for H2′ NMR resonance suggested that –OH groups in the ribose are the binding sites for Sb(V) probably via ring chelation at C2′ and C3′.

The complexation of Sb(V) with ribonucleosides was found to be faster at acidic pH, indicating that it is kinetically favored in acidic biological compartments [60,61]. Another remarkable property of these complexes is their very slow dissociation rate constant in aqueous solutions at neutral pH [60]. Moreover, the value of stability constant determined for 1:1 Sb(V)-GMP complex [60] is consistent with the formation of such complex in the vertebrate host following treatment with pentavalent antimonial drugs, especially if one considers the high accumulation and prolonged retention of antimony in macrophages [62]. With respect to the possible pharmacological role of Sb(V)-ribonucleosides complexes, two hypotheses may be raised [60]. Such complex might act as an inhibitor of the *Leishmania* purine transporters. Alternatively, these complexes might penetrate inside the parasite, encountering a neutral pH-environment and then interfere with the purine salvage pathway, like the purine analog, allopurinol [63]. The formation of these complexes may also explain the depletion of ATP and GTP, as reported previously after exposition of *Leishmania* parasite to sodium stibogluconate [64,65].

It has been suggested that the mode of action of pentavalent antimonials is also dependent on a number of factors including T-cell subsets and cytokines [66]. Stibogluconate was found to be a potent inhibitor of protein tyrosine phosphatases, which leads to an increase in cytokine responses [67]. Another recent study revealed that meglumine antimoniate increased the phagocytic capacity of monocytes and neutrophils and enhanced superoxide anion production by phagocytes, which represent the first line of defense against the parasite [68]. These results taken altogether suggest that Sb(V) may kill the parasites by both direct and indirect mechanisms, the host response being implicated in the activity of Sb(V).

Finally, a promising strategy for improving the efficacy of antimonial chemotherapy involves the association of pentavalent antimonials with immunomodulators. So far, this approach was found to be effective in reducing the applied dose of antimonial, while maintaining the treatment efficacy [69,70].

## Synthetic Processes for Pentavalent Antimonials

Two processes proposed in a Rhône Poulenc patent [71,72] for the synthesis of meglumine antimoniate start either from SbCl_5_ or from SbCl_3_. More recently, new synthetic methods for preparation of pentavalent organoantimonials have been described [26,73,74,75]. Two of these methods used SbCl_5_ as a source of Sb(V) [73,75] and another one used KSb(OH)_6_ [74].

Interestingly, the pentavalent compound obtained from KSb(OH)_6_ contained less than 0.0015% (w/w) of residual Sb(III) [76]. This amount of Sb(III) was more than 10-fold lower than those found in the compound prepared from SbCl_5_ and in different commercial lots of meglumine antimoniate [76]. The compounds obtained from KSb(OH)_6_ and SbCl_5_ were evaluated *in vitro* and *in vivo* on *L. amazonensis* infections. Although *in vitro* the most effective drugs contained the highest levels of Sb(III), no correlation was found *in vivo* between the antileishmanial activity of meglumine antimoniate and its residual Sb(III) content, suggesting that residual Sb(III) contributes only marginally *in vivo* to the drug antileishmanial activity [76]. Importantly, the synthetic compounds showed *in vivo* anti-leishmanial efficacies similar to that of the commercial drug. It should be mentioned that even though residual Sb(III) did not affect the drug antileishmanial effectiveness, it may be responsible for some of the side effects of the pentavalent antimonial drug. In this context, strategies to reduce the amount of residual Sb(III) in pentavalent antimonial drugs may result in safer treatments. These new processes may encounter application in the industrial production of meglumine antimoniate and result in improved quality and reduced cost of the final product.

## Liposome-based Formulations

The use of liposomes has been so far one of the most efficient means for improving antimonial effectiveness against visceral leishmaniasis. At least four different properties make liposomes the most appropriate carrier system for antimonials: (i) their ability to effectively encapsulate and retain large amounts of water-soluble compounds [77]; (ii) their natural tendency to be captured by the macrophages of the reticuloendothelial system, which are the same cells that harbor *Leishmania* parasites; (iii) their relative safety; (iv) their high versatility with respect to lipid composition, volume and composition of internal compartment, vesicle size and lamellarity.

The impact of liposome encapsulation on the anti-leishmanial activity of antimonial drugs was first reported in the eighties, following investigation in mice, hamsters and dogs experimentally infected with *Leishmania donovani* [78,79,80]. On the basis of parasite suppression in the liver and/or spleen, liposome-encapsulated meglumine antimoniate and sodium stibogluconate administered intravenously were more than 700 times more active compared to either of the free (unencapsulated) drugs. This spectacular effect of liposome encapsulation was attributed to the marked targeting of antimony to infection sites [81]. It was anticipated that liposomes would improve antimonial therapy, allowing for the reduction of applied dose and of the frequency of dosing. Thus, both reduction of metal-related side effects and enhancement of drug effectiveness can be expected.

The complexity of liposomal drug formulations, when compared to conventional drugs, implies that not only pharmacological issues but also technological problems related to their production should be considered. In that sense, a critical point is the choice of the method of preparation of the liposome-based formulation. Until recently, two different methods were used for the encapsulation of antimonial drugs in liposomes. One method consists of the hydration of a thin film of lipids with a solution of the drug [82]. The other method, known as reverse-phase evaporation procedure, involves the formation of a water-in-oil emulsion using the drug solution as aqueous phase followed by evaporation of the organic solvent, which results in a phase change and the formation of a vesicle suspension [83]. The main advantage of the latter method, compared to the former, is that it yields higher efficiencies of drug encapsulation and higher ratios of encapsulated drug to lipid. These characteristics mean that a lower quantity of lipid has to be injected in order to introduce the same quantity of antimonial, which makes the treatment safer and more economical. Nevertheless, liposomes prepared by the reverse-phase evaporation procedure may be toxic at high doses due to unavoidable residual traces of organic solvent in the final liposome formulation. Another significant limitation of these methods is that the resulting liposome preparations could be stored only as aqueous suspensions. In this condition, however, a significant leakage of the drug occurred with time from the internal aqueous phase into the external continuous aqueous phase. For instance, a typical liposomal formulation prepared by the reverse-phase evaporation procedure released more than 26-48% of the originally encapsulated drug when stored for 7 weeks at 25 ºC [83].

**Figure 3:**
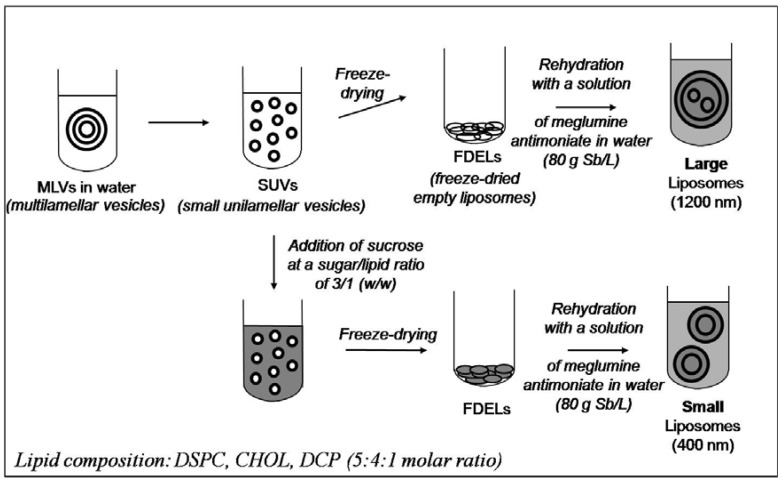
Processes used for the preparation of meglumine antimoniate-containing liposomes of different size. Adapted from Ref. [84,85,86,87].

Our group recently described novel liposomal formulations of meglumine antimoniate, which were obtained through rehydration of freeze-dried empty liposomes with an aqueous solution of the antimonial compound [84,85,86,87]. Figure 3 displays the processes used to obtain these formulations. A significant technological advantage of this method over conventional ones [93,94] is that liposomes may be stored as pre-formed freeze-dried empty vesicles and that rehydration may be performed just before use. Two different liposomal formulations, with mean hydrodynamic vesicle diameters of 1,200 nm and 400 nm, were obtained [84]. 

Since dogs infected with *Leishmania chagasi* or *infantum* are the main natural reservoir of visceral leishmaniasis in Latin America and in the Mediterranean region, but respond poorly to conventional anti-leishmanial therapies, much effort has been devoted to the achievement of an effective liposome formulation in these animals [84,88,89,90,91,92,93,94]. This context also stimulated our group to investigate the pharmacokinetics and therapeutic efficacy of our large and small sized vesicles formulations in this animal model. 

The liposome formulation of meglumine antimoniate consisting of large sized vesicles was first evaluated in dogs with visceral leishmaniasis [93,95]. Following multiple dose-regimen (four doses of 4 mg Sb/kg of body weight with 4-days intervals), this formulation resulted in a significantly lower number of positive dogs (compared to the group of dogs treated with empty liposomes or that of untreated dogs), but was unable to clear *Leishmania* *chagasi* parasites in the bone marrow, suggesting that this tissue may be critical for the treatment with this liposomal formulation.

**Figure 4:**
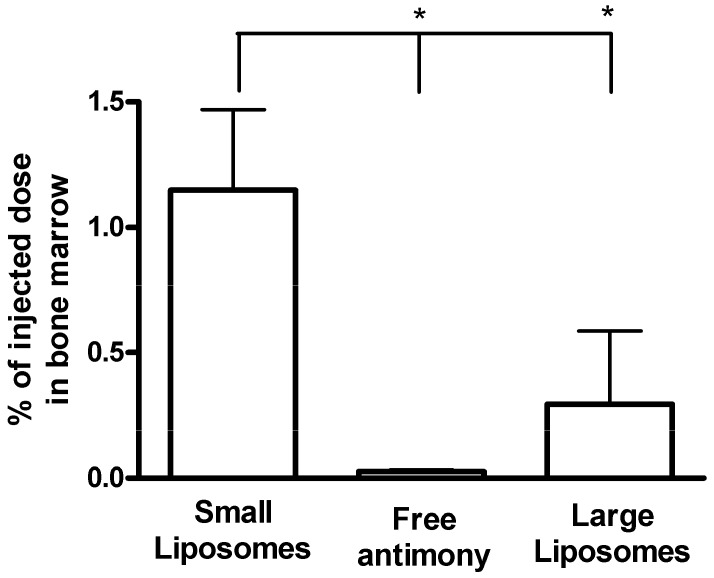
Influence of liposome encapsulation and of vesicle mean diameter on antimony level in the bone marrow of infected dogs, 96 h after intravenous bolus injection of meglumine antimoniate. Small liposomes (mean vesicle diameter of 400 nm) were given at 4 mg Sb/kg of body weight (n = 5). Large liposomes (mean vesicle diameter of 1,200 nm) were given at 5.5 mg Sb/kg of body weight (n = 10). Meglumine antimoniate was given at 100 mg Sb/kg of body weight (n = 5). Data are given as means ± standard deviation. *P <0.001 for One-way ANOVA followed by Bonferroni post-test. Adapted from Ref. [84].

In a recent study, the pharmacokinetics of the formulation of meglumine antimoniate in small sized vesicles (mean hydrodynamic diameter of 400 nm) was evaluated in mongrel dogs naturally infected with *Leishmania chagasi* [84]. Following intravenous administration at 4 mg Sb/kg, antimony was rapidly cleared from the circulation with two phases with half-lives of about 10 and 45 min. Four days after injection, 38%, 8% and 1.2% of the injected dose of antimony were found in the liver, spleen and bone marrow, respectively. As illustrated in Figure 4, this formulation was found to target antimony to the bone marrow of dogs at a 3-fold higher level, when compared to the formulation of meglumine antimoniate in large sized liposomes [84], suggesting that it may clear more effectively parasites from this tissue.

In a subsequent study, the antileishmanial activity of the formulation of meglumine antimoniate in small sized liposomes was investigated in naturally-infected dogs [94]. Immunocytochemical evaluations of cervical lymph nodes, liver, and spleen of dogs for *Leishmania* parasites, 5 months after treatment with four doses of 6.5 mg Sb/kg of liposomal meglumine antimoniate, showed parasite suppressions higher than 95% when compared to control groups, either untreated or treated with empty liposomes. Feeding of *Lutzomyia longipalpis* phlebotomines on dogs treated with liposomal meglumine antimoniate, five months after treatment, resulted in a significant reduction of sand fly infection efficiency, compared to feeding on control animals. This study was the first report of both long-term parasite suppression and reduction of infectivity to sand flies in naturally infected dogs following treatment with a liposome-encapsulated drug. Importantly, this was achieved using a 20-fold-lower cumulative dose of Sb than is used for conventional antimonial treatment. Nevertheless, despite these very promising results, the dosing regimen used in this study was unable to clear *Leishmania* parasites in the bone marrow of treated dogs.

Safety evaluations in dogs treated with the liposome formulations indicated no change in serum markers of hepatic function (aspartate aminotransferase, alkaline phosphatase, alanine aminotransferase, total bilirubins) and renal function (urea, creatinine). The hemogram parameters also did not show any significant alteration upon treatment [94]. On the other hand, transitory adverse reactions, including prostration, defecation, tachypnea, and sialorrhea were observed during the first 15 min after each injection of the liposome formulations. Such lipid vesicle-induced acute effects have been described previously as complement-mediated pseudoallergic reactions [96] and should be reduced by using a drug infusion instead of bolus injection.

Further improvement of the efficacy of liposomal treatment is expected from the increase of the duration of treatment, the use of liposomes of even smaller size, the combination of liposomal Sb with other antileishmanial agent or the combination of liposomal Sb with an immunomodulators.

It is noteworthy that chemotherapy in dogs with visceral leishmaniasis is not allowed in Brazil, especially with antimonial drugs, mainly because dogs are the main reservoir and such treatment may lead to generate drug resistance. Thus, a novel liposome formulation and dosing regimen, in order to be applicable to the clinical treatment of infected dogs, should block the transmission of parasites to the sand fly without inducing drug resistance. Although this point still requires investigation, one can hypothesized that liposomal therapy, when compared to conventional therapy, may reduce the risk of appearance of drug resistance, by promoting a very high drug concentration at the target starting from the first dose.

It is often considered that the relatively high cost of phospholipids used to prepare liposomes represents a major obstacle to the development of liposomal formulations. However, it is important to point out that, in the specific case of antimonial formulation, the cost of treatment with the liposomal drug would not be necessarily higher than that of conventional antimonial therapy. In a liposome-based treatment, much lower amount antimony would be used and the cost would be determined mainly by lipids. Assuming that the cost of lipids is about US$ 10 per g (based on the price of lipids in Lipoid GmbH catalogue) and that of Glucantime® is about US$ 1.2 per 5 mL-vial (http://www.essentialdrugs.org/edrug/archive/200705/msg00062.php), a four-dose treatment with 6.5 mg Sb/kg of liposomal meglumine antimoniate would be (0.7-fold) cheaper than a conventional treatment with 20 doses of 30 mg Sb/kg (recommended for dog treatment).

## Cyclodextrin-based Formulations

Conventional pentavalent antimonials cannot be administered orally due to their poor absorption and/or inactivation in the stomach, and their parenteral delivery requires a multiple-dose regimen because of their rapid renal clearance [10,11]. Taken together, these problems lead to non-compliance of the dose regimen and consequently drug resistance. It is therefore desirable to develop methods for enhancing the bioavailability of antimonials by oral route.

The association of drugs to carrier systems is a feasible strategy to improve their oral absorption. Cyclodextrins, which are cyclic oligosaccharides composed of glucose units joined through α-1,4 glucosidic bonds, are well known in recognition chemistry as molecular hosts capable of including, with a degree of selectivity, water-insoluble guest molecules via non-covalent interactions within their hydrophobic cavity. Thus, this carrier has been widely used to improve the oral bioavailability of water-insoluble drugs, owing to the enhanced drug solubility and dissolution rate [97,98].

Demicheli and coworkers reported recently the preparation of a meglumine antimoniate-β-cyclodextrin (β-CD) composition, through heating of an equimolar mixture of meglumine antimoniate and β-CD in water, followed by freeze-drying [99].

Importantly, the association of meglumine antimoniate with β-CD enhanced the absorption of Sb by the oral route and rendered the antimonial drug orally-active in a murine model of cutaneous leishmaniasis [100]. The anti-leishmanial activity was evaluated in BALB/c mice experimentally infected with *Leishmania amazonensis*. Animals orally treated with the meglumine antimoniate/β-CD composition (daily doses of 32 mg Sb/kg for 12 days) developed significantly smaller cutaneous lesions when compared to those treated with saline or higher doses of meglumine antimoniate (daily oral doses of 120 mg Sb/kg for 12 days). Strikingly, the effectiveness of the composition given orally was equivalent to that of meglumine antimoniate given intraperitoneally with a 2-fold higher dose of antimony. The anti-leishmanial activity of the complex was confirmed by the significantly lower parasite load in the lesions of treated animals, when compared to saline controls [100]. In these *in vivo* assays, no sign of acute toxicity has been observed. 

This study established, for the first time, the potential of cyclodextrin-based formulation for the oral treatment of leishmaniasis with meglumine antimoniate [99,100]. Importantly, it presents the first orally-active formulation for a pentavalent antimonial drug.

In fact, the ability of β-CD to enhance the absorption of meglumine antimoniate by the oral route is quite surprising, considering that meglumine antimoniate is highly water-soluble (> 300 mg/ml) and that this cyclodextrin classically acts by improving the solubility of poorly water-soluble drugs. This led us to investigate the mode of action of this non-conventional drug-cyclodextrin complex. Physicochemical characterization by ESI-MS and circular dichroism revealed that several factors may account for the improved oral absorption of antimony. First, heating of the meglumine antimoniate + β-CD mixture was found to induce the depolymerization of meglumine antimoniate from high molecular weight Sb complexes into 1:1 Sb-meglumine complex, as well as the formation of a new ternary meglumine-Sb-β-CD complex [101]. Secondly, the freeze-drying step promoted additional associations between antimony and β-CD, including the formation of 1:2:1, 2:2:1 and 2:2:2 meglumine-Sb-β-CD complexes, which resulted in supramolecular nanoassemblies with a mean hydrodynamic diameter of about 200 nm [102]. Another important observation was the ability of the meglumine antimoniate/β-CD composition to act as a sustained release system of the antimonial drug [102], suggesting that this property may result in the prolongation of the drug absorption in the gastrointestinal tract. 

**Figure 5:**
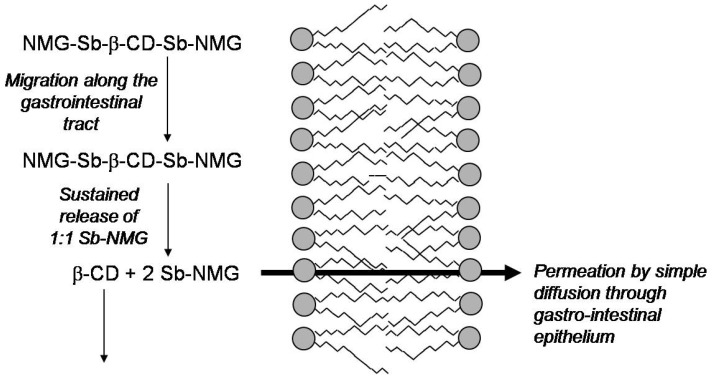
Model proposed for the mechanisms involved in the enhanced absorption of Sb from oral meglumine antimoniate/β-CD composition. The meglumine antimoniate/β-CD nanoassemblies comprising high-molecular weight meglumine antimoniate/β-CD complexes, such as NMG-Sb-β-CD-Sb-NMG species, migrate along the gastrointestinal tract. These nanoassemblies then slowly release meglumine antimoniate in the form of 1:1 Sb-NMG complex which permeates by simple diffusion across the gastrointestinal epithelium. β-CD continue migrating up to the colon where it is degraded. Adapted from Ref. [102].

Figure 5 illustrates the model proposed for the enhanced delivery of Sb from the meglumine antimoniate/β-CD composition by the oral route. Accordingly, the slow release property of meglumine antimoniate /β-CD nanoassemblies would allow for their migration along the gastrointestinal tract. These nanoassemblies would then release meglumine antimoniate in the form of 1:1 Sb-meglumine complex, which would readily permeate by simple diffusion across the intestinal epithelium.

In conclusion, these studies provide the first experimental evidence that the oral bioavailability of pentavalent antimonial drugs can be improved, through the formation of labile covalent complexes, involving Sb(V) and the hydroxyl groups of a biodegradable carrier.

## Conclusions

During the present decade, progress has been achieved towards the improvement of antimonial chemotherapy. Recent developments include new insights into the structure and mechanisms of action of pentavalent antimonials, some novel synthetic methods for preparation of these compounds, pharmaceutically-acceptable liposome-based formulations for targeting *Leishmania* parasites responsible for visceral leishmaniasis and a β-cyclodextrin-based formulation for promoting the oral delivery of antimony. These recent achievements reveal new directions for the improvement of antimonial chemotherapy.
